# Lactate in skin homeostasis: metabolism, skin barrier, and immunomodulation

**DOI:** 10.3389/fimmu.2025.1510559

**Published:** 2025-02-19

**Authors:** Dandan Ruan, Tingting Hu, Xuefan Yang, Xiaohui Mo, Qiang Ju

**Affiliations:** Department of Dermatology, Renji Hospital, Shanghai Jiao Tong University School of Medicine, Shanghai, China

**Keywords:** lactate, metabolism, skin barrier, immunomodulation, therapeutics

## Abstract

Lactate, once considered merely a byproduct of glycolysis, is now increasingly recognized as a multifunctional signaling molecule with roles beyond energy metabolism. It functions as an enzyme cofactor and binds to specific receptors to modulate cellular functions. In the skin, lactate is produced by various cell types. It is then transferred between cells or to the extracellular space, helping to balance cellular pH and to provide signals that regulate skin barrier and skin immunity. Additionally, lactate/lactate-related genes hold promising therapeutic potential for the treatment of skin tumors, inflammatory skin diseases, hair loss, and in cosmetic dermatology. This article highlights the latest advances in our understanding of lactate’s biological effects on the skin and explores its therapeutic potential, offering insights into future research directions.

## 1 Introduction

Historically regarded as a metabolic byproduct, lactate is recently recognized as a pleiotropic signaling molecule, with diverse roles in regulating immune-inflammatory response, angiogenesis, tumorigenesis, and metabolic regulation (1, 2). In the context of skin physiology, lactate has emerged as a key player in maintaining skin homeostasis and is potentially involved in various skin diseases. Understanding the precise mechanisms by which lactate influences skin biology is crucial for advancing this field.

Since the first discovery in 1927 that the skin can convert glucose to lactate, numerous studies have confirmed the presence of essential metabolic pathways including glycolysis, the tricarboxylic acid (TCA) cycle, and the hexose monophosphate (HMP) pathway in the skin (3). Despite the presence of mitochondria in skin cells, such as epidermal and sebaceous gland (SG) cells, glycolysis is the predominant pathway, with most glucose being converted to lactate (4, 5). A study has shown that lactate concentrations in the skin are significantly higher than those in plasma (6). The lactate produced in the skin can enter systemic circulation, where it serves as a substrate for gluconeogenesis in the liver, thereby contributing to overall glucose homeostasis. This process, often referred to as the skin’s Cori cycle, which highlights the skin’s active role in metabolic regulation (3). Beyond its function as an energy substrate, lactate plays a crucial role in maintaining skin homeostasis and modulating immune responses. A recent study has shown that lactate, through the lactate/GPR81 pathway, can alleviate imiquimod-induced psoriasis (7). However, excessive lactate production in the epidermis can diffuse into the dermis, where it modulates immune cell function and potentially exacerbates inflammatory skin diseases (8, 9).

Despite its emerging significance, lactate’s role in skin barrier function, immune regulation, and disease progression remains underexplored. This article reviews recent advances in the understanding of lactate’s biological impact on skin physiology and pathology, with particular emphasis on its contributions to skin barrier function and immunomodulation. The aim is to provide a theoretical framework for the potential application of lactate or lactate-associated genes in cosmetics interventions and clinical treatments.

## 2 Lactate production and skin-specific metabolism of lactate

### 2.1 Lactate production in skin: pathways and cellular contributions

**Figure 1:**
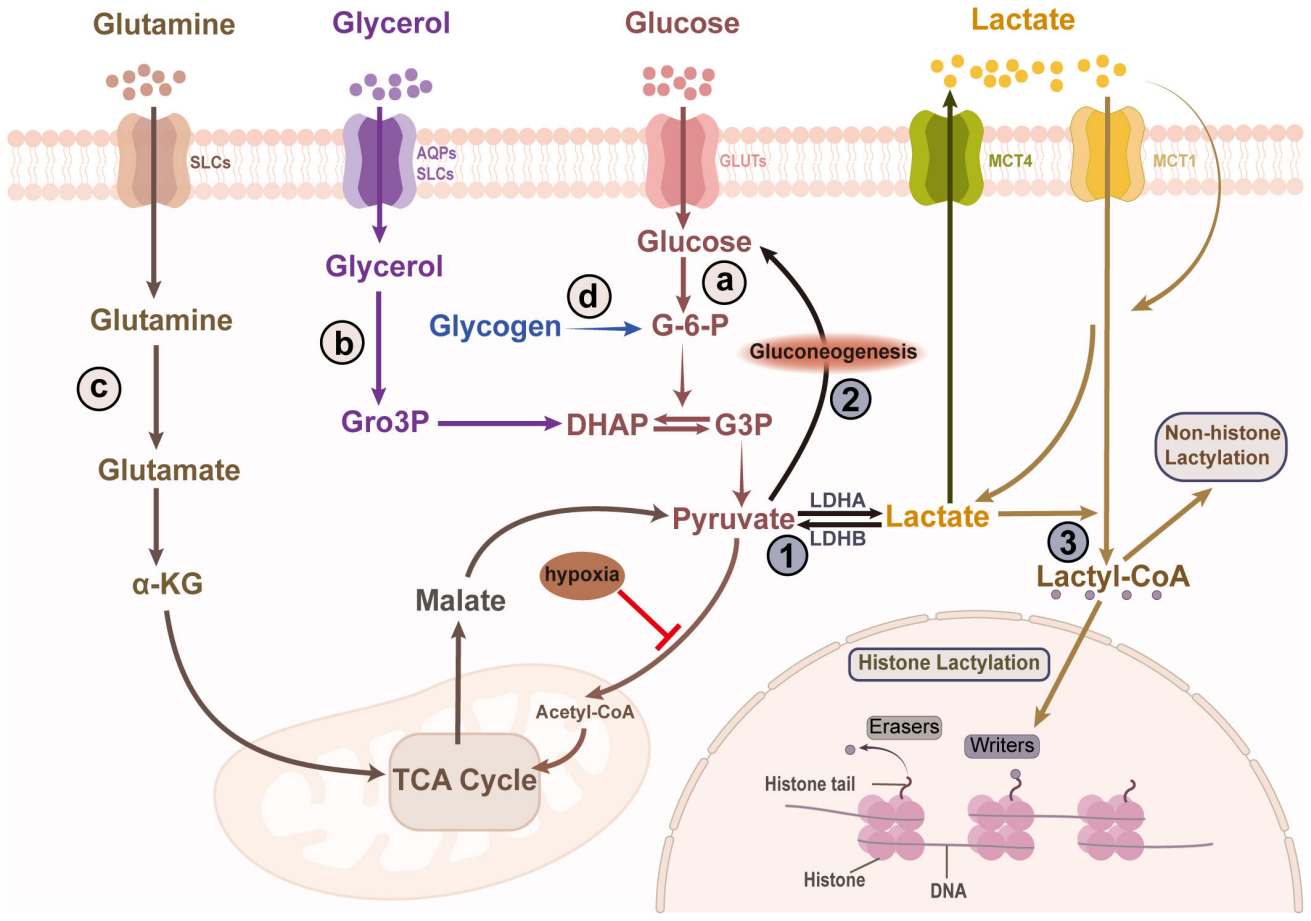
Skin lactate production and clearance. Lactate in the skin is produced through the breakdown of various substrates, including glucose, glycogen, glycerol, and glutamine. The four sources of lactate are as follows: **(A)** Lactate is a main byproduct of glycolytic metabolism. Pyruvate, generated via glycolysis, is reduced to lactate by LDHA. **(B)** Glycerol is converted to Gro3P by glycerol kinase, which is then converted to DHAP by α phosphoglycerol dehydrogenase, ultimately entering glycolysis to produce lactate. **(C)** Glutamine, via glutaminase, is converted to glutamate, which undergoes deamination to produce α-ketoglutarate. This metabolite enters the TCA cycle, generating pyruvate, which is subsequently converted into lactate. **(D)** Lactate can also be produce from glycogen via glycogenolysis. Lactate has three major fates in the skin cells. Another situation is that lactate is transported out of the cell, which is also a way to clear lactate. ①It is converted to pyruvate, which enters mitochondria and participates in the TCA cycle. ②Although the epidermis lacks the key enzyme for gluconeogenesis, the hair follicle can convert lactate into glucose through gluconeogenesis which is stored as glycogen. ③Lactate participates in protein lactylation by being transformed into lactyl-CoA. LDHA/B, lactate dehydrogenase A/B; SLCs, solute carriers; AQPs, aquaporins; GLUTs, glucose transporters; MCT1/4, monocarboxylate transporter 1/4; Gro3P, glycerol-3-phosphate; DHAP, dihydroxyacetone phosphate; G-6-P, Glucose 6-phosphate; G3P, glyceraldehyde 3-phosphate; TCA, tricarboxylic acid; lactyl-CoA, lactoyl-coenzyme A.

The skin is a major source of lactate, with various skin cells—including sebocytes, keratinocytes, and hair follicle stem cells—demonstrating a remarkable capacity for lactate production, resulting in elevated lactate concentrations in the skin compared to those in the serum (4, 6, 10, 11). The primary substrates for lactate production in the skin include glycogen, glucose, glutamine, and glycerol. Lactate is predominantly generated through glycolysis, in which glucose undergoes a series of enzymatic reactions that culminate in the production of lactate. Glycogen, as a storage form of glucose, can be converted to glucose-6-phosphate, which then enters the glycolytic pathway. Glycerol can be converted to glycerol-3-phosphate by glycerol kinase, which is further metabolized into dihydroxyacetone phosphate by glycerol-3-phosphate dehydrogenase (12). This dihydroxyacetone phosphate then feeds into glycolysis, ultimately contributing to lactate formation. Additionally, glutamine catabolism serves as another significant source of lactate (1). In the presence of glutaminase, glutamine is first converted to glutamate, which then undergoes deamination to produce α-ketoglutarate. This molecule enters the TCA cycle generating pyruvate, which is subsequently converted to lactate (**Figure 1**).

**Figure 2:**
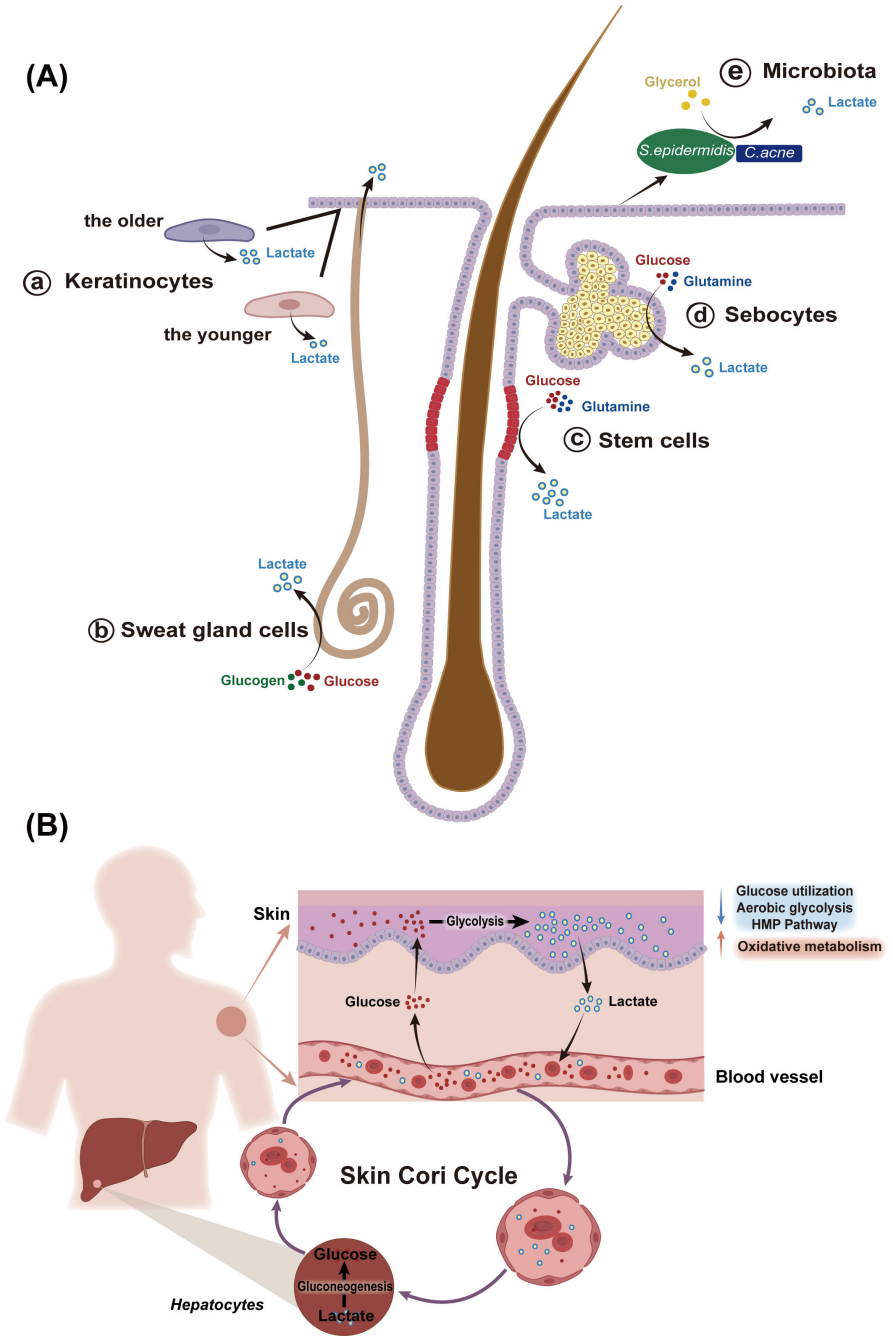
Sources of lactate in the skin. **(A)** The skin is an important source of lactate, with various skin cells, especially human hair follicle stem cells, keratinocytes, and sebocytes, producing lactate at levels higher than those in the serum. (a) Keratinocytes have a strong ability to generate lactate *in vitro*, with increased lactate generation observed in aged keratinocytes compared to those from younger individuals. (b) Sweat glands in the dermis and hypodermis also produce lactate, which is excreted through sweat onto the skin surface. (c) Human scalp hair follicles preferentially engage in glycolysis followed by lactate production in the presence of oxygen. Hair follicle stem cells can produce significantly more lactate than other epidermal cells. (d) sebaceous glands, a major source of skin lipids, also generate lactate, relying on glycolysis and glutaminolysis. (e) *C. acnes* and *S.epidermidis*, which colonize the skin surface and appendages also generate lactate during the process of decomposing metabolic substances. *S.epidermidis, Staphylococcus epidermidis; C.acnes, Cutibacterium acnes.* **(B)** The concept of skin Cori cycle elucidates the skin’s important role in carbohydrate metabolism. the epidermis converts large amounts of glucose to lactate, which is released into the bloodstream and serves as a substrate for gluconeogenesis in the liver. Conversely, glucose from the blood can be temporarily stored in the dermis and transferred to the epidermis. Moreover, the pattern of glucose metabolism in the epidermis changes with keratinocytes differentiation: as keratinocyte differentiation from the basal to the granular layer, the activities of glucose utilization, aerobic glycolytic, and the HMP pathway decreases, while mitochondrial oxidative metabolism increases. HMP, hexose monophosphate.

The activity of these metabolic processes is cell-specific (**Figure 2A**). Keratinocytes, for example, have a notable capacity to generate lactate *in vitro (* 11). Interestingly, lactate production in keratinocytes is increased in the elderly, a pattern also observed in aged fibroblasts (13, 14). Hair follicle stem cells primarily rely on glycolytic metabolism, producing more lactate than other epidermal cells, even in the presence of oxygen (10, 15). Sweat gland cells, located in the dermis and hypodermis, also produce lactate, which can be secreted to the skin surface via sweat (16). SGs, as an important source of skin lipids, have been reported to be glycolytic and glutaminolytic tissues, converting a substantial portion of glucose to lactate even under aerobic conditions (4). Moreover, the skin microbiota, including *Cutibacterium acnes, Staphylococcus epidermidis*, and *lactic acid bacteria*, contribute to lactate production through their own metabolic processes (17–19). Lactate exists as two optical isomers: L-lactate and D-lactate. Due to the lack of D-lactate dehydrogenase in humans, L-lactate is the predominant form involved in human biological processes (20). However, D-lactate can still be detected in the human body, often considered a bacterial metabolite produced by the microbiota (20). Recent studies have suggested that D-lactate can be endogenously synthesized in humans via the methylglyoxal pathway (20). Given the distinct biological roles of these lactate isomers and the diverse skin microflora, understanding the ratio of L-lactate to D-lactate in the skin could provide valuable insights into skin physiology and inform potential therapeutic strategies for skin diseases.

Importantly, lactate in the skin is not merely a byproduct of metabolism. It plays an active role in various metabolic pathways. Lactate can be converted back to pyruvate, which then enters the mitochondria to participate in the TCA cycle for further oxidation and decomposition. Additionally, lactate can be converted to glucose through gluconeogenesis, however, this pathway may be absent in the epidermis due to the lack of key enzymes (3). Interestingly, outer root sheath keratinocytes in human hair can synthesize glycogen from lactate, suggesting that gluconeogenesis pathways may be present in these cells (21). This may be related to the unique cyclic changes and microenvironments within hair follicles. Furthermore, lactate can serve as a precursor for lactyl-CoA, participating in the modification of histones and non-histone proteins, thereby influencing gene expression and cellular functions (1) (**Figure 1**).

In conclusion, the diverse metabolic pathways of lactate—including glycolysis, gluconeogenesis, and histone modification—highlight its critical role in maintaining skin homeostasis. Understanding the balance and functions of lactate in the skin is crucial for advancing our knowledge of skin physiology and treating skin-related diseases.

### 2.2 Metabolic characteristics of lactate in skin

After exploring the pathways of lactate production within the body, it is crucial to recognize the skin as a significant source of lactate. The epidermis displays a high rate of glycolysis, a low level of TCA cycle activity, and abundant LDH enzymes, leading to substantial lactate production (22, 23). This metabolic profile positions the skin as a key player in the body’s overall lactate dynamics, similar to skeletal muscles. The concept of a “skin Cori cycle” has been proposed, suggesting that lactate produced by skin cells can enter the bloodstream, be transported to the liver, and subsequently converted into glucose, thereby contributing to the body’s carbohydrate metabolism (3) (**Figure 2B**). Additionally, lactate distribution in the skin shows spatial variation; for example, lactate concentration in the forearm is more than three times higher than in the upper arm (24).

**Figure 3:**
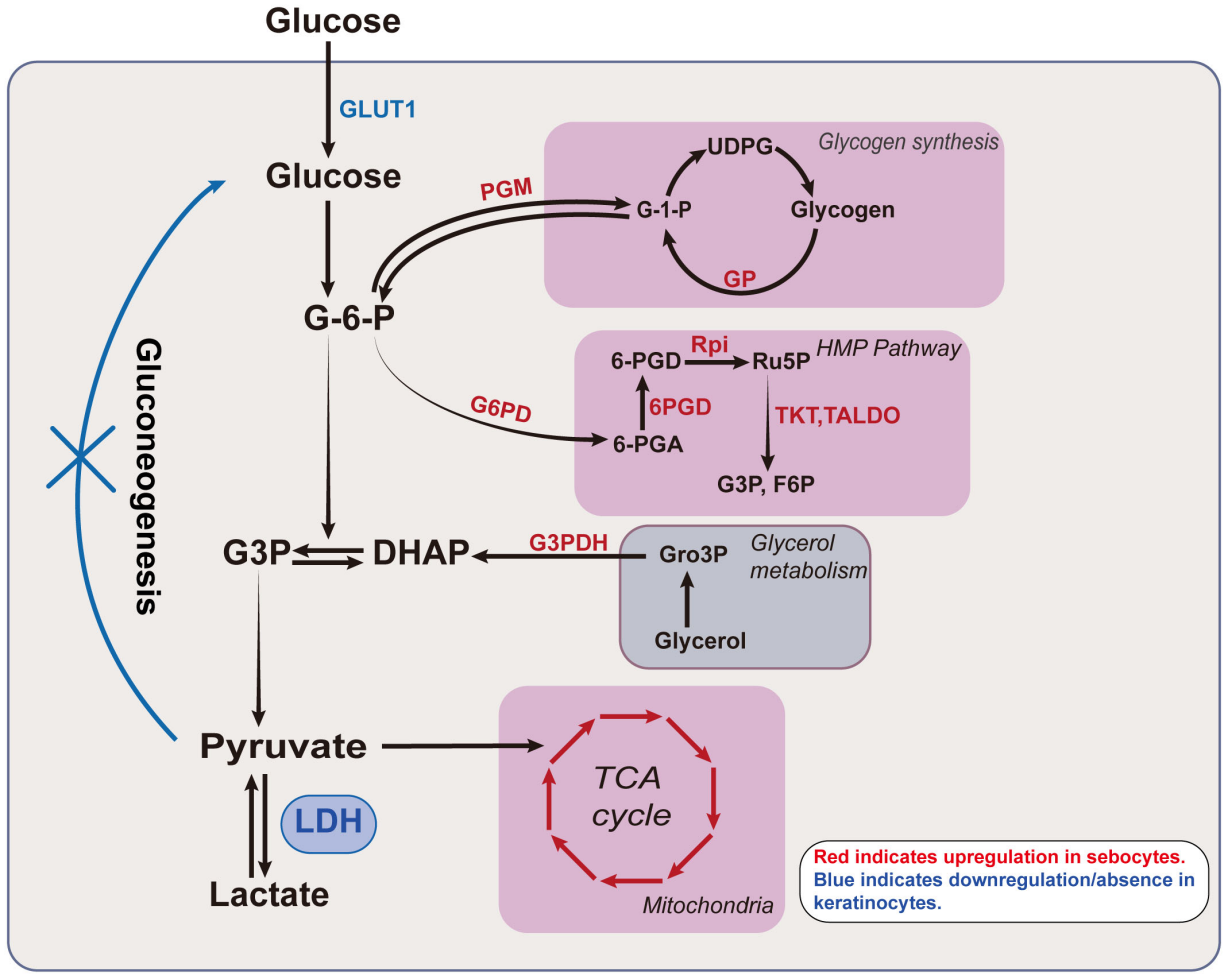
Lactate related metabolism in keratinocytes and sebocytes. Glucose undergoes various enzymatic reactions to ultimately produce lactate. The intermediates in this process link glycolysis to other metabolic pathways. For example, G-6-P can enter glycogen metabolism and the HMP pathway, while DHAP connects glycerol metabolism with lactate production. The classical TCA cycle connects glutamine metabolism with glycolysis via pyruvate. The activity of different glucose metabolic pathways is cell-specific and exhibits differentiation in keratinocytes and sebocytes. The activity of various enzymes in sebocytes, including those involved in the TCA cycle, glycogen metabolism, glycerol metabolism, and the HMP pathway is markedly higher than in epidermal keratinocytes, with enzymes such as PGM, GP, G6PD, 6PGD, Rpi, TKT, TALDO and G3PDH showing significantly elevated activity in sebocytes. In contrast, epidermal keratinocytes exhibit higher expression of GLUT1 and LDH. The red letters/arrows indicate upregulated enzymes/pathways in sebocytes, while the blue letters/lines denote the downregulated/or absent metabolic pathways in keratinocytes. GLUT1, glucose transporter type 1; G-6-P, glucose-6-phosphate; G3P, glyceraldehyde 3-phosphate; DHAP, dihydroxyacetone phosphate; LDH, lactate dehydrogenase; G-1-P, Glucose 1-phosphate; UDPG, uridine diphosphate glucose; 6-PGA, 6-phosphogluconic acid; 6PGD, 6-phosphogluconate dehydrogenase; Ru5p, ribulose 5-phosphate; F6P, fructose-6-phosphate; HMP, hexose monophosphate; Gro3P, glycerol-3-phosphate; TCA, tricarboxylic acid; PGM, phosphoglucomutase; GP, glycogen phosphorylase; G6PG, glucose-6-phosphate dehydrogenase; Rpi, ribose 5-phosphate isomerase; TKT, transketolase; TALDO, transaldolase; G3PDH, glycerol-3-phosphate dehydrogenase.

At the tissue level, both the epidermis and SGs are capable of lactate production, yet they differ in glucose metabolism (3, 23, 25–27). The functional metabolic profile is cell-specific, reflecting variations in enzyme activity and substrate availability that regulate metabolic efficiency (**Figure 3**). For instance, as epidermal cells differentiate and mature, their glucose metabolism patterns shift. During keratinocyte differentiation from the basal to the granular layer, overall glucose utilization, HMP activity, and aerobic glycolytic activity progressively decrease, while mitochondrial oxidative metabolism increases. This reflects an enhancement in mitochondrial efficiency and a shift in cellular function (22). In parallel, an increase in the number of mitochondrial clusters is also observed as differentiation progresses, suggesting that keratinocytes rely on the mitochondrial function to meet their metabolic demands during mid-differentiation stages before mitochondria diminish at higher levels of keratinization (28). In contrast, in SGs, as cells mature from the periphery towards the center, a decrease in glucose, glycogen, Adenosine 5’-triphosphate(ATP), and glycolytic enzyme levels is noted (27).

These observations raise the question: why do skin cells produce such high levels of lactate under physiological conditions? Functionally, this high lactate production is likely associated with the active synthesis of proteins and lipids, as well as the continuous renewal of skin cells. Glycolysis provides rapid energy for biosynthetic processes. In SGs, for example, intermediates from glycolysis, such as pyruvate, lactate, glycerol-3-phosphate, and reduced nicotinamide adenine dinucleotide (NADH), play dual roles. Not only do they serve as precursors for lipid synthesis but also contribute essential energy and reductive power through alternative metabolic pathways, including the TCA cycle and the HMP pathway (29).

In summary, the high production of lactate in skin cells underscores lactate’s role in skin energy metabolism and reveals its multifaceted roles in maintaining skin health and function. A deeper understanding of processes provides valuable insights into skin physiology and may inform potential therapeutic strategies for skin diseases.

## 3 Lactate as a signaling molecule

### 3.1 Molecular mechanisms of lactate signaling and lactate transporters

Lactate, beyond its classical role as a metabolic byproduct, plays a pivotal role in cellular communication. Its transport between cells occurs predominantly through monocarboxylate transporters (MCTs) or via free diffusion. It acts as a signaling molecule, engaging G protein-coupled receptors (GPRs) to modulate cellular behavior. Recent studies have identified lactate as a ligand for GPR68, GPR81, and GPR132, also known as ovarian cancer G protein-coupled receptor 1 (OGR1), hydroxy-carboxylic acid receptor 1 (HCAR1) and G2 accumulation protein (G2A), respectively (30–34) (**Table 1**). These receptors are sensitive to changes in pH, and their activation is triggered at specific pH values, linking lactate signaling to microenvironmental pH variations (30–34). Furthermore, lactate can also influence cell functions through post-translational modification (PTM). Lactylation, a newly identified modification, is one such mechanism by which lactate mediates its effects on the biological function of cells.

**Table 1:** Expression of lactate-related receptors and transporters in skin cells.

	GPR68	GPR81	GPR132	MCT1	MCT4
Keratinocytes	–	–	+^1^	+^2^	+^3^
Fibroblasts	+^3^	+^4^	+^3^	+^5^	+^6^
Macrophages	+^7^	+^8^	+^9^	+^10^	+^11^
Mast cells	–	–	–	+^12^	–
Sebaceous glands	–	–	–	+^13^	–
Hair follicles	–	–	–	+^14^	–

”+”, Expressed; “-”, no data.

PMIDs of reference (1–14) : 27623507, 38973262, 25461841, 37653539, 39102921, 29809274, 28174749, 33123172, 38012414, 36268709, 38733581, 27559047, 19048272, 33548088.

GPR68, G protein-coupled receptor 68; GPR81, G protein-coupled receptor 81; GPR132, G protein-coupled receptor 132; MCT1, Monocarboxylate transporter 1; MCT4, Monocarboxylate transporter.

#### 3.1.1 MCTs

The transport of lactate across cell membranes is primarily mediated by MCTs, which enable bidirectional flux depending on lactate concentration and local pH changes (35–37). In skin cells, MCTs play crucial roles in regulating lactate uptake and efflux. MCT1/4 are the principal transporters expressed in various skin cells, including keratinocytes, macrophages, and fibroblasts (8, 38, 39). In the epidermis, MCT1 is predominantly expressed in the suprabasal layers, facilitating the uptake of lactate (38), While MCT4 is expressed throughout the epidermis, with higher levels in the suprabasal layers, mediating lactate efflux (38). In human scalp hair follicles, MCT1 is enriched in the apical membrane of bulb epithelial cells and in the most proximal outer root sheath of the intra-bulb region (15). In SGs, MCT1 expression gradually decreases from the glandular portion towards the excretory ducts, with no expression in fully mature cells (40, 41).

MCTs play a critical role in the regulation of skin inflammation, immune function, and collagen synthesis in the dermis. For instance, the expression of MCT1 is upregulated in skin inflammatory disorders, such as psoriasis, contributing to the metabolic and immune dysregulation observed in these conditions (38). Inhibition of MCT1 and MCT4 has been shown to mitigate inflammatory responses by reducing lactate-induced macrophage matrix metalloproteinase (MMP) release, suppressing NF-κB pathway activation, and subsequently reducing extracellular matrix degradation (8). Moreover, blocking MCT1 can inhibit glycolytic activity and Interleukin(IL)-9 release in T helper(Th) 9 cells, modulating inflammation in allergic skin diseases (40). In skin allografts, MCT1 inhibition impairs T cell proliferation, thereby reducing the acute rejection responses (41). Importantly, MCT1 inhibition also diminishes histone lactylation in fibroblasts, leading to reduced collagen synthesis and indicating its role in extracellular matrix remodeling (39).

#### 3.1.2 GPRs

Lactate acts as an endogenous ligand for GPR81, a receptor primarily involved in regulating inflammation (42, 43). Although GPR81 is not detected in skin-specific cells, such as keratinocytes, it has recently been identified in skin immune cells, including macrophages and dendritic cells (44). Studies involving GPR81 knockout or inhibition have demonstrated its critical role in inflammation regulation, fibrosis, angiogenesis, and hematopoiesis (45–49). Notably, GPR81 mediates lactate-induced anti-inflammatory signaling in chronic inflammatory skin diseases. Loss of GPR81 function can promote macrophage polarization towards the pro-inflammatory M1 phenotype and prolong activation of the NF-κB pathway, exacerbating the psoriasis-like phenotype in mice (7). A recent study has demonstrated that lactate has the capacity to suppress tumor necrosis factor(TNF)-α-induced MMP-9 expression and prevent the disruption of tight-junction proteins by inhibiting NF-κB p65 activation through GPR81 (50).

In addition to GPR81, lactate also signals through other GPRs, such as GPR68 and GPR132. GPR68 is expressed in various cells, including monocytes/macrophages, T cells, granulocytes, and skin fibroblasts (51, 52). This receptor regulates inflammation in a ligand-specific manner (30). Its knockout or inhibition can mitigate fibrogenesis and inhibit acid-induced lipogenesis (53, 54). The role of GPR132 in lactate is less well understood, but studies have shown that GPR132 is expressed in the human epidermis, keratinocytes, fibroblasts, melanocytes, and various skin tumors, such as malignant melanoma, compound nevus cell nevi, and basal cell carcinoma (51, 55, 56). GPR132 signaling has been reported to be involved in skin immune regulation and cell cycle arrest (32, 34). Deletion of GPR132 reduces lactate-induced M2 macrophage polarization and inhibits lysophosphatidylcholine-mediated glycolytic activity and inflammatory factor expression in keratinocytes (57, 58).

#### 3.1.3 PTMs

As a byproduct of glucose metabolism, lactate exerts its biological effects through PTMs, particularly lactylation and acetylation, which are important modifications associated with gene transcriptional regulation (59–61). Lactate is reported to promote global lactylation and histone H3 lysine 18 lactylation (H3K18lac) levels in HaCaT cells *in vitro* (62). Lactylation presents in normal skin and is altered in certain pathological conditions, including melanoma and psoriasis (62, 63). In cutaneous melanoma, calmodulin-like 5 (CALML5) has been identified as a core lactylation-associated gene, and the expression of which is significantly lower than that in normal skin tissues (64). While another study has shown up-regulated lactylation in ocular melanoma, the inhibition of which efficiently suppressed the tumor progression, indicating a novel therapeutic target for ocular melanoma (63). Lactate has been shown to trigger latent-transforming growth factor beta-binding protein 1 (LTBP1) lactylation at lysine 752, thereby promoting skin rejuvenation through the induction of collagen synthesis in fibroblasts (39). Additionally, lactate is an endogenous histone deacetylase inhibitor (HDACI), which can influence acetylation and deacetylation (61). These processes have been shown to play important roles in skin diseases such as psoriasis and in skin aging (65, 66). Future studies are required to investigate the precise role of lactate in PTMs of skin and the related immune cells, as well as the interaction among the involved PTMs.

### 3.2 Regulation of skin barrier by lactate

**Figure 4:**
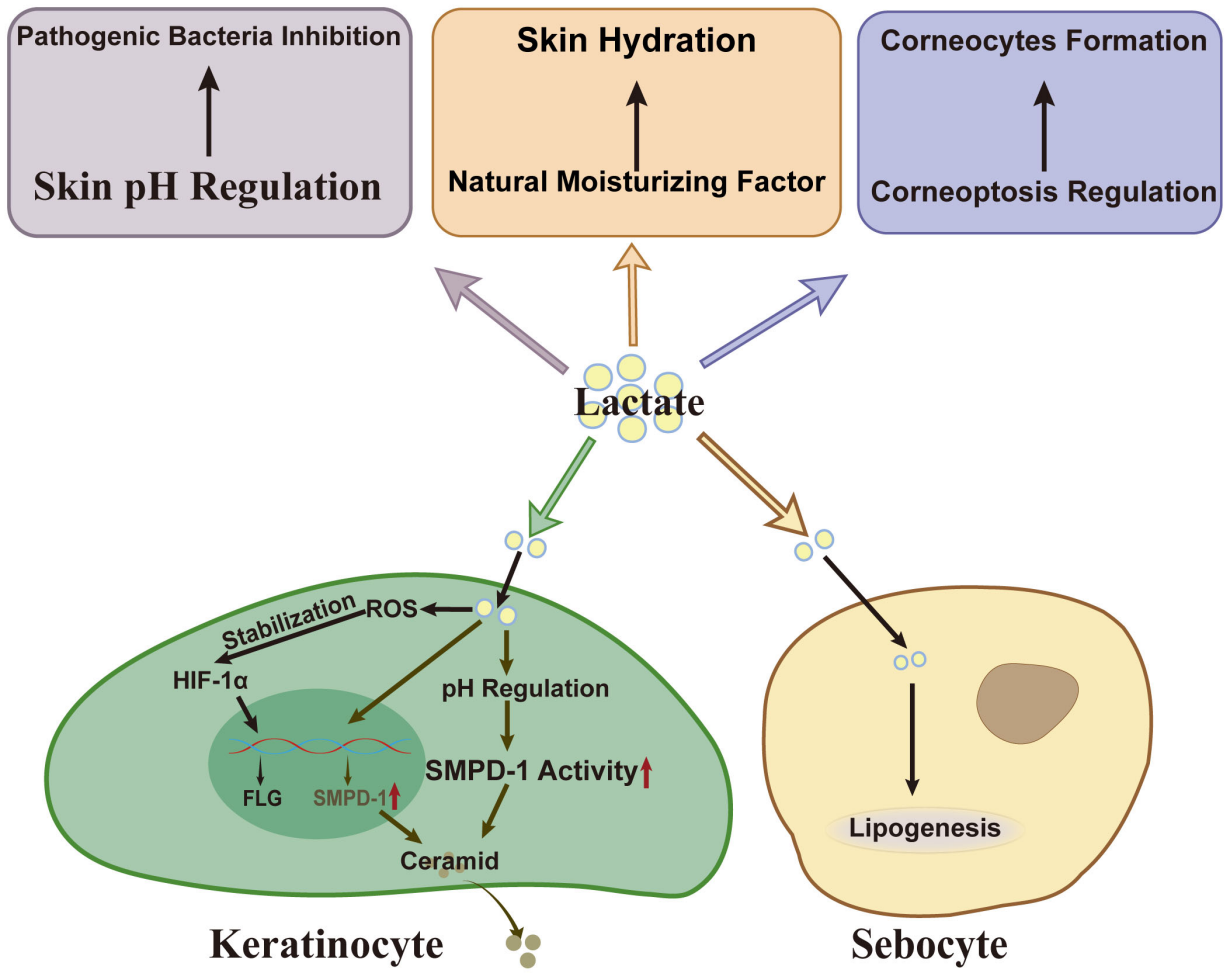
Lactate’s effect on skin barrier. As a weak acid, lactate inhibits the growth of *S.aureus* and *C.acnes*. Besides, lactate and its metabolic pathways influence corneocytes formation by modulating corneoptosis, a specific form of programmed cell death in the stratum corneum. Lactate also serves as a natural moisturizing factor (NMF), essential for maintaining skin hydration. In keratinocytes, lactate enhances filaggrin gene transcription by stabilizing HIF-1α. Lactate also upregulates SMPD-1 expression or activity via pH regulation, facilitating ceramid synthesis. In sebocytes, lactate acts as a substrate in regulating lipogenesis. *S.aureus, Staphylococcus aureus;* ROS, reactive oxygen species; HIF-1α, hypoxia-inducible factor-1 alpha; SMPD-1, sphingomyelin phosphodiesterase-1; FLG, filaggrin.

The skin, as the outermost organ of the human body, is composed of the dermis and a stratified keratinized epithelium that undergoes terminal differentiation and lifelong renewal. It serves as a critical barrier, protecting underlying tissues and organs from external harmful factors, while also maintaining overall homeostasis of the body. The stratum corneum, consisting of protein-rich cells and lipid-rich intercellular domains, functions primarily as a physical barrier (67). Additionally, the skin’s acidic pH contributes to a chemical barrier, inhibiting pathogen invasion and maintaining the balance of the skin microbiome (68). Lactate regulates the expression of related proteins and enzymes in keratinocytes, particularly through its involvement in acidification and corneoptosis, thus influencing the skin’s barrier function (**Figure 4**).

#### 3.2.1 Lactate regulation of filaggrin expression, ceramide synthesis, and skin hydration

Lactate has been shown to up-regulate the expression of filaggrin in HaCaT cells and 3D skin models, enhancing the ability of keratinocytes to synthesize ceramides (18, 69). Under normoxic conditions, hypoxia-inducible factor (HIF)-1α is typically hydroxylated and degraded, limiting its biological activity (70). Interestingly, lactate can stabilize HIF-1α even under normoxic conditions, increasing its abundance in a reactive oxygen species-dependent manner (71). This stabilization enhances HIF-1α’s ability to promote the transcription of key barrier-related genes, such as filaggrin by binding to hypoxia response elements (HREs) in their promoter regions (72). In addition, HIF-1α regulates glycolysis in skin cells, leading to lactate accumulation (73). The interplay between lactate and HIF-1α not only maintains HIF-1α activity but also highlights the reciprocal regulation between lactate dynamics and transcriptional control, supporting the expression of key barrier-related genes and maintaining skin barrier integrity.

In addition to its effects on protein expression, lactate also regulates skin lipids, such as ceramides, and the hydration of the stratum corneum, further underscoring its crucial role in maintaining skin barrier homeostasis. Lactate may promote ceramide biosynthesis through several mechanisms. For instance, in hairless mouse models, lactate enhances the synthesis and secretion of skin lamellar bodies, which are rich in various lipids and enzymes associated with ceramide production (74). Furthermore, lactate, as an organic acid, can regulate skin pH, helping to maintain the optimal acidic environment for the high activity of ceramides synthesis-related enzymes such as sphingomyelin phosphodiesterase-1 (75). *In vitro*, lactate fermented with glycerol promotes the expression of sphingomyelin phosphodiesterase-1 (18). Moreover, lactate itself acts as a natural moisturizing factor, absorbing moisture from the environment and increasing the hydration of the stratum corneum, thereby contributing to its flexibility and integrity (18). Numerous clinical studies have shown that lactate enhances stratum corneum hydration and increases epidermal thickness (76, 77). Notably, lactate also serves as a precursor for lipid synthesis in SGs, influencing the rate of lipogenesis (78).

#### 3.2.2 Lactate regulation of skin pH and corneoptosis

Recent studies have revealed that the pH of the human stratum corneum is stratified into three distinct layers: a moderately acidic lower layer, an acidic middle layer, and a pH-neutral upper layer (79). This stratified pH profile is essential for the barrier function of the stratum corneum. As a weak acid with a pKa of 3.86, lactate can modulate skin pH and influence cellular functions by acidifying the cell microenvironment (80). At the surface of the stratum corneum, lactate inhibits the growth of *Staphylococcus aureus* and *Cutibacterium acnes* (18, 81).

Beyond its direct role in pH regulation, lactate, and its metabolic pathways may also influence corneocyte formation by modulating corneoptosis, a form of programmed cell death specific to the stratum corneum (82). During the differentiation of granular layer cells into corneocytes, intracellular calcium (Ca^2+^) levels rise, and rapid acidification occurs, facilitating the clearance of organelles (82). A potential link exists between elevated intracellular calcium concentrations and the plasma membrane Ca2+ ATPase (PMCA), an ion pump responsible for extruding calcium ions from the cell (83). PMCA function relies on ATP produced through glycolysis (83). In the skin, PMCA4 expression increases from the basal layer to the granular layer, while the glycolysis rate in the epidermal cells decreases as they differentiate (84). The ATP generated may become insufficient to sustain PMCA activity, leading to elevated intracellular calcium levels and triggering corneoptosis. Interestingly, although glycolysis is downregulated in the granular layer, lactate produced in the lower layer may enter the keratinocytes in its undissociated form, further acidifying the cells and aiding organelle clearance, which is crucial for corneoptosis.

### 3.3 Bidirectional regulation of skin immunoinflammation by lactate

**Figure 5:**
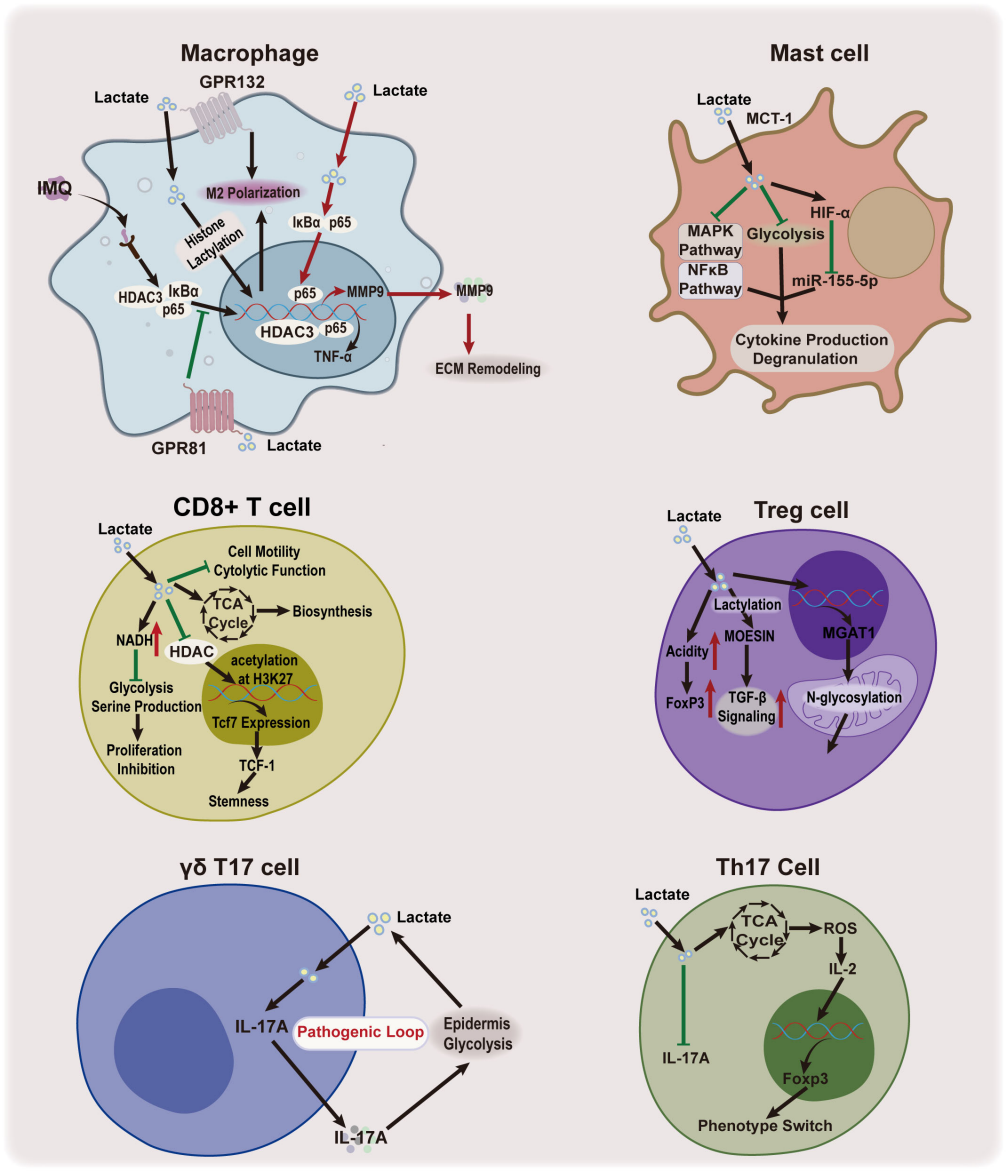
Lactate’s effect on skin immunoinflammation. Lactate plays a dual role in regulating inflammatory immune response. It functions as both a pro-inflammatory agent and an anti-inflammatory mediator, influencing macrophages and IL-17-producing cells, while it an anti-inflammatory property in mast cells. Lactate has also been shown to enhance the function of Treg cells. IMQ, imiquimod; HDAC3, histone deacetylase 3; TNF-α, tumor necrosis factor alpha; GPR81, G protein-coupled receptor81; GPR132, G protein-coupled receptor 132; MCT1, monocarboxylate transporter 1; MAPK, mitogen-activated protein kinase; HIF-1α, hypoxia-inducible factor-1 alpha; miR-155-5p, microRNA-155-5p; Th17 cell, IL-17-producing T helper cell; IL-17A, interleukin 17A; TCA, tricarboxylic acid; ROS, reactive oxygen species; IL-2, interleukin 2; Foxp3, Forkhead/winged helix transcriptional factor P3; MMP9, matrix metallopeptidase 9; ECM, extracellular matrix; NADH, Nicotinamide adenine dinucleotide; Tcf7, T cell factor 7; TCF-1, T cell factor-1;Treg cell, regulatory T cell; H3K27, Histone H3 Lysine 27;TGF-β, Transforming growth factor beta; MOESIN, membrane-organizing extension spike protein; OXPHOS, oxidative phosphorylation; MGAT1, alpha-1,3-mannosyl-glycoprotein 2-beta-N-acetylglucosaminyltransferase.

Lactate, the end product of glycolysis, serves not only as a marker of cellular metabolic activity but also plays a pivotal role in regulating immune response and inflammation in the skin. Studies in oncology have shown that lactate can promote angiogenesis and immune escape (1). However, the lactylation-induced macrophage polarization, accompanied by the expression of homeostatic genes, has been shown to exhibit anti-inflammatory properties (1). Lactate’s dual role in modulating the cutaneous inflammatory immune response highlights its potential as a key regulator in balancing immune and inflammatory pathways, offering therapeutic value for inflammatory and immunological skin diseases (**Figure 5**).

#### 3.3.1 Macrophages

Macrophages, the most abundant immune cells in the skin, exhibit significant plasticity, transitioning between pro-inflammatory(M1) and anti-inflammatory(M2) phenotypes in response to stimuli (85). Recent studies have demonstrated that lactate plays a pivotal role in this process. It can induce M2 polarization by promoting protein lactylation or activating receptors such as GPR132 (57, 86–88). Additionally, lactate regulates the NF-κB signaling cascade— exogenous lactate, through GPR81 activation prevents IκBα degradation and inhibits the nuclear translocation of HDAC3 and p65 in macrophages, thereby reducing inflammatory cytokine production and improving psoriasis in mouse models (7). A recent study has shown that lactate can reduce mitochondrial antiviral signaling protein (MAVS) and downstream NF-κB activation through retinoic acid-inducible gene 1 (RIG-I) lactylation (89). The lactylation of RIG-I reduces NLR family pyrin domain containing 3 (Nlrp3) activation and induces M2 polarization in macrophages (89). Moreover, lactate can suppress pro-inflammatory cytokine production in macrophages by inhibiting the activation of yes-associated protein(YAP) and NF-κB via GPR81-mediated signaling (90). In addition, epidermal-derived lactate serves as a metabolic substrate for dermal macrophages, activating the NF-κB pathway and the TCA cycle to promote M2 polarization and increase matrix metalloprotease 9(MMP9) expression (8). Although lactate supports tissue remodeling, excessive MMP9 can degrade the basement membrane and promote skin inflammation (8). In a mouse model of imiquimod-induced psoriasis, inhibiting MCT4 with the MCT1/4 blocker, syrosingopine, partially alleviated psoriasis by disrupting the lactate-p65-NF-κB-MMP9 axis (8). These findings collectively emphasize lactate’s context-dependent regulatory effects on macrophage function and skin inflammation, suggesting a complex role that warrants further research to optimize its therapeutic potential.

#### 3.3.2 Mast cells

Mast cells, which originate from the myeloid lineage, reside in skin tissue and play a crucial role in inflammatory and allergic skin diseases (91, 92). Lactate has been shown to regulate mast cell function, with studies indicating that it suppresses IgE- and Mas-related G protein-coupled receptor X2-mediated activation, including cytokine production and degranulation, in response to lipopolysaccharide and IL-33 (93–96). Most of these inhibitory effects depend on pH or MCT1 expression (93–96). Mechanistic studies have shown that lactate inhibits the phosphorylation of the mitogen-activated protein kinases(MAPK)(e.g. extracellular signal-regulated kinase, c-Jun N-terminal kinases) and NF-κB (e.g. p65) signaling pathways in mast cells, which are associated with inflammation (93, 94). Lactate also inhibits the rate of mast cell glycolysis and downregulates microRNA-155-5p via HIF-1α, further inhibiting cytokine release (94, 95). Taken together, these research suggest that lactate acts as an immune regulator that modulates mast cell ability, limiting their contribution to inflammation in various pathological conditions.

#### 3.3.3 IL-17-producing T cells

IL-17 is a major inflammatory factor elevated in several skin inflammatory diseases, including psoriasis, atopic dermatitis(AD), and hidradenitis suppurativa (97). Elevated IL-17 levels are primarily produced by neutrophils, Th17 cells, mast cells, CD8^+^ T cells, αβ T, γδ T cells, and innate lymphoid cells (98). The balance between Th17 and regulatory T (Treg) cells is the key for regulating IL-17 production and maintaining immune homeostasis. An imbalance, particularly a shift towards Th17 cells, exacerbates inflammation, as observed in hidradenitis suppurativa with an increased Th17/Treg ratio (99). Lactate influences this Th17/Treg balance, modulating IL-17 production through several mechanisms. Lactate inhibits CD4+ T cell-derived Th17 cell proliferation and phenotype, reducing the IL-17 production (100, 101). Moreover, lactate promotes metabolic and epigenetic reprogramming of pro‐inflammatory Th17 cells, facilitating their conversion into Treg cells, which further suppresses inflammation (101). This shift is critical for controlling excessive inflammation and promoting immune tolerance. Lactate also enhances Treg cell function and generation (102). One primary pathway is the modulation of MOESIN lactylation, which facilitates interaction with the transforming growth factor beta (TGF-β) receptor, enhancing TGF-β signaling and SMAD3 activation. and enhancing TGF-β signaling (103). Additionally, lactate supports Treg cell metabolism via α-1,3-Mannosyl-Glycoprotein 2-β-N-Acetylglucosaminyltransferase(MGAT1)-mediated N-glycosylation in mitochondria, contributing to local microenvironment acidification and stabilizing Treg cell populations, maintaining their immunosuppressive functions (102, 104).

In addition to its effects on Th17 and Treg cells, lactate also influences γδ T17 cells, which are tissue-resident T cells and key producers of IL-17 in the dermis (9, 105). Lactate produced by abnormal glycolysis in the epidermis permeates into the dermis and promotes IL-17 production by γδ T17 cells (9). Subsequently, IL-17 exerts its effects on the cells at the lesion site, further promoting glycolysis and establishing a critical pathogenic feedback loop (9). This process is likely mediated by lactate’s role in regulating oxidative phosphorylation in γδ T17 cells, which are dependent on mitochondrial oxidative phosphorylation for cytokine production (106). Lactate, as an energy substrate, plays a crucial role in stimulating cellular oxidative phosphorylation, and enhancing cellular energy production (2). CD8+ T cells, another source of IL-17, exhibit a more complex, and context-dependent response to lactate (107). Lactate has been shown to inhibit CD8+ T cell proliferation by modulating the NAD(H) redox state, thereby affecting their metabolic activity (108). Moreover, lactate can impair cytolytic function and reduce cell motility (109). However, under specific conditions, lactate enhances the stemness of CD8 + T cells and boosts their bioenergetic and biosynthetic capacities by acting as a substrate for the TCA cycle (110, 111). These findings suggest that lactate exerts a nuanced regulatory effect on immune responses, modulating immune cell functions in response to the tissue microenvironment.

Lactate’s effects on skin immune cells are multifaceted, with both pro-inflammatory and anti-inflammatory properties. Given its inhibitory effects on mast cell-induced inflammation, topical lactate administration may hold therapeutic potential for acute skin inflammation. However, further research is needed to fully understand the conditions under which lactate’s dual effects can be harnessed for therapeutic interventions.

### 3.4 Lactate as a potential therapeutic target and agent

Lactate, which accumulates in physiological conditions, is further elevated in numerous skin disorders, including both malignant and non-malignant skin tumors, inflammatory diseases, and skin damage, such as diabetic wounds. Specifically, lactate levels are significantly increased in lesions of melanoma and squamous cell carcinomas of the head and neck (HNSCC) (112–114). Elevated glucose metabolism and lactate production have also been observed in keloid fibroblasts (115). Furthermore, enrichment of HIF-1α signaling, as well as glycolysis and gluconeogenesis pathways, has been identified in human skin lesions from conditions such as psoriasis, AD, and hidradenitis suppurativa, indicating active metabolic and signaling alterations (9). In AD patients, serum samples show elevated anaerobic glycolysis-derived lactate, accompanied by reduced levels of β-oxidation metabolites (26). Additionally, decreased lactate levels in the natural moisturizing factor from the stratum corneum of mild AD patients correlate with the elevated skin pH typically observed in these individuals (116). Increased lactate levels of the wound fluid are detected in non-infected diabetic wounds and are even higher in infected ones (117).

Due to the free shuttle of lactate between cells and tissues, varying lactate concentrations in different skin regions may reflect its specific physiological role. Notably, distinct gene expression patterns related to lactate dynamics—such as MCT1, MCT4, and glucose transporter type 1(GLUT-1)—have been observed in human scalp hair follicles, further supporting lactate’s functional significance in skin tissues (15). With the growing recognition of lactate as a signaling molecule, new clinical perspectives and treatment strategies have emerged, particularly in the context of targeting lactate and its related genes in skin tumors. In addition, lactate’s potential therapeutic applications extend to inflammatory skin diseases, hair loss, wound healing, and skin aging. Recent advancements in topical formulations or related delivery systems containing lactate components have become a focal point of research.

#### 3.4.1 Skin tumor

In melanoma, lactate is primarily derived from circulating lactate and is regulated by MCTs (112). Specifically, MCT1 levels are elevated in highly metastatic melanoma, and inhibition of MCT1 can reduce lactate uptake, indicating that lactate plays a critical role in melanoma metastasis (112). Additionally, lactate has been shown to induce the expressions of vascular endothelial growth factor (VEGF) and IL-8, both of which promote angiogenesis in melanoma cells, thereby supporting tumor growth (118, 119). Recent studies have shown that lactate promotes the polarization of macrophages towards an M2 phenotype, which subsequently enhances the expression of VEGF, TGF-β, and IL-10 (120). These factors, in turn, facilitate angiogenesis and contribute to melanoma progression (120). Furthermore, lactate can suppress the function and survival of T cells and NK cells, contributing to tumor immune evasion in melanoma (118). In ocular melanoma, elevated lactylation has been detected, and the inhibiting lactylation efficiently suppresses tumor progression (63). The recent study has shown that melanoma-derived lactate can activate sterol-regulatory element-binding protein (SREBP)-2, driving tolerogenic dendritic cell maturation and promotes melanoma progression (121). In HNSCC, lactate concentration is positively correlated with resistance to fractionated irradiation (122). The enzyme LDH5 is highly expressed in HNSCC, and its levels are associated with local recurrence, survival, and distant metastasis, indicating its role as a marker of radiation resistance and a potential therapeutic target (123). Lysine lactylation (Kla) has been detected in SCC25 cells, with significant differences observed between normoxic and hypoxic conditions (124). Moreover, a negative correlation between tissue Kla levels and the prognosis in oral SCC patients has been revealed by immunohistochemistry (124). The deletion of sirtuin 6 (SIRT6) has been shown to enhance lactate production in HNSCC, promoting a more aggressive tumorigenic phenotype (125).

Lactate, glycolysis-related genes, and lactate-dependent post-translational modifications (PTMs) play crucial roles in the growth, metastasis, and treatment resistance of skin tumors. Targeting lactate and its associated pathways offers a promising valuable therapeutic strategy for skin tumors, including melanoma and squamous cell carcinoma (SCC). For example, glycolysis inhibition due to human rhomboid family-1(RHBDF1) deficiency has been reported to enhance the efficacy of immunotherapy in murine melanoma models (126). Recent studies have also identified stiripentol, an LDHA inhibitor, as a potential therapeutic agent (127). Stiripentol reduces DNA repair efficiency by preventing lactylation of Nijmegen breakage syndrome protein 1(NBS1) at lysine 388, thereby overcoming chemotherapy resistance (127). Additionally, recently, poly (lactic-co-glycolic) acid (PLGA)-based nanoparticles combining photothermal therapy with epigenetic therapy have been shown to slow tumor progression and improve median survival in a syngeneic murine melanoma model (128). Moreover, a lactate oxidase-based nanocapsule enzyme therapeutic has been demonstrated to prevent tumor immunosuppression and improve the efficacy of immune checkpoint blockade treatment in a murine melanoma model (129).This approach offers a promising strategy for improving cancer immunotherapy.

#### 3.4.2 Skin chronic inflammatory disorders

Lactate’s biological effects are modulated by various factors, including the route of administration, dosage, and the specific site of action. These observations highlight the complexity of lactate and its potential therapeutic value in inflammatory skin diseases. For instance, oral administration of lactate has been shown to alleviate psoriatic symptoms in mouse models, whereas subcutaneous injection elicits an opposing effect (7, 8). This highlights the dependence of lactate’s mechanisms on its mode of delivery and the specific cellular microenvironment. Further investigation into these mechanisms is crucial to elucidate lactate’s role in the pathogenesis of inflammatory skin diseases, providing a solid foundation for developing targeted therapeutic interventions (7, 8). In pathological states, excessive cutaneous lactate production and altered energy metabolism contribute to the progression of inflammatory diseases. Consequently, strategies aimed at inhibiting lactate production or modulating its metabolic pathways are being explored as promising therapeutic approaches. This hypothesis is supported by evidence that metformin, a glycolysis inhibitor, reduces the proliferation of keratinocytes and Th17 cells, as well as cytokine production in hyperproliferative inflammatory skin diseases (130, 131). Interestingly, lactate itself has been shown to inhibit glycolysis by downregulating key enzymes, such as hexokinase and 6-phosphofructo-1-kinase (132, 133).

While inhibiting glycolysis presents a potential strategy for managing chronic inflammatory skin diseases, lactate itself may serve as an alternative therapeutic agent. Clinical evidence supports the use of lactate, *lactic acid bacteria*, and yogurt in treating psoriasis, AD, and acne (7, 134, 135). Recent studies have also demonstrated that exogenous lactate application exerts anti-inflammatory effects by modulating immune cell behavior, restoring skin barrier function, and promoting tissue repair. For example, oral administration of lactate has been shown to reduce the severity of psoriasis-like skin lesions by decreasing excessive keratinocyte proliferation and immune cell infiltration (7). Topical lactate, as a natural moisturizing compound, has been demonstrated to relieve symptoms of dry skin, maintain skin barrier integrity, and exhibit antioxidant properties (136). Furthermore, lactate acts as an epidermal exfoliant, facilitating skin cell renewal and improving dermatological symptoms (137). Advances in topical carrier technology, particularly polylactic acid (PLA)-based formulations, have enhanced therapeutic efficacy while reducing side effects (138). Notably, lactate derived from PLA degradation can function as a ligand for GPR68, inhibiting the activation of NF-κB p65 and p38 mitogen-activated protein kinase, which in turn modulates the secretion of pro-inflammatory cytokines (30). Topical application of hydrogel films loaded with pyruvate and lactate has also shown promise in mitigating skin inflammation and oxidative stress induced by ultraviolet radiation (139). Additionally, lactate’s role in epigenetic modification warrants further investigation. Lactate influences gene expression and exerts anti-inflammatory effects through the modulation of histone lactylation and by inhibiting the activity of HDACs. HDAC inhibition promotes terminal differentiation of keratinocytes, enhancing the expression of filaggrin and transglutaminase-1, while inhibiting IL-33 production (140, 141). Interestingly, reduced levels of global lactylation, H3K18lac, and adiponectin have been reported in the skin tissues of psoriasis patients (62). Lactate treatment has been shown to upregulate lactylation levels and promote adiponectin expression in HaCaT cells, which is linked to its anti-inflammatory effect (62).

#### 3.4.3 Alopecia

The proliferation and activation of hair follicle stem cells are crucial for the progression of the hair cycle. Lactate and LDH-related genes play key roles in the activation of hair follicle stem cells. Specifically, deletion of LDHA in hair follicle stem cells prevents their activation while lactate induction accelerates their activation and the hair cycle (10). Furthermore, the c-Myc/LDHA axis has been demonstrated to induce metabolic reprogramming, driving hair follicle stem cell proliferation and differentiation (142). Metabolic programs in human anagen hair follicles have been proposed due to the distinct expressions of related genes in human scalp hair follicles (15). Additionally, lactate is converted to glycogen within human outer root sheath keratinocytes (21, 143). This resultant elevation in glycogen levels has been linked to hair growth (21). A sufficient blood supply is essential for hair follicle growth and development. An *in vitro* experiment has demonstrated that lactate promotes the production of VEGF, which stimulates angiogenesis around hair follicles by acting on the endothelial cells of dermal vessels (77, 144). This, in turn, provides nutrition to hair follicles, supporting hair growth.

Clinically, while current treatments for alopecia include topical minoxidil, oral finasteride, and oral baricitinib, targeted therapies focused on LDH-related metabolic pathways have emerged as a promising area of research (145). A recent innovation in this field involves a separable microneedle patch composed of chitosan lactate and exosomes derived from adipose stem cells (146). This microneedle patch has demonstrated superior efficacy in promoting hair regeneration compared to topical minoxidil in animal models, while also exhibiting antibacterial properties (146). Such drug-free, transdermal microneedle patches represent a safe and efficient strategy for alopecia treatment, with great potential for clinical application.

#### 3.4.4 Wound healing

Lactate is a crucial substance in restoring the integrity of the skin barrier under pathological conditions, contributing to wound healing through several key mechanisms. First, as an energy substrate, lactate meets the elevated metabolic demands of healing tissues (147). Second, accumulated lactate reduces the pH in wounds (147). This acidification facilitates cellular proliferation and differentiation within the optimized physiological pH range (147). Third, lactate promotes angiogenesis and stimulates fibroblasts to synthesize collagen in the extracellular matrix, thereby aiding tissue regeneration (148).

Recent studies have demonstrated that the c-MYC/LDHA axis drives metabolic reprogramming, proliferation, and differentiation of hair follicle stem cells, suggesting that lactate may also enhance wound healing by affecting stem cell properties (142). Additionally, lactate produced by *Lactobacillus reuteri* has been shown to accelerate wound healing by altering the wound environment, inhibiting the CD26 enzyme, and increasing the bioavailability of CXCL12 (149). Lactate interacts with mitochondria and ROS, influencing growth and collagen expression in human fibroblasts (150). However, excessive lactate concentrations can impair cell viability in fibroblasts and endothelial cells, underscoring the need to carefully monitor lactate levels in wound fluid (151). Thus, determining the optimal lactate concentration in wound fluid before treatment is crucial. Advancements in non-invasive lactate detection technology, which can monitor lactate levels in skin interstitial or wound fluid, are equally important for clinical applications (152). Clinically, poly (lactic-co-glycolic acid) (PLGA), a well-established drug delivery system, has been explored as a vehicle to promote wound healing by delivering therapeutic drugs, such as antibiotics and anti-inflammatory drugs (153).

The investigation of lactate’s role in wound healing not only highlights its significance in metabolic regulation, immune response, and tissue repair, but also paves new avenues for clinical therapy and material innovation. As research into lactate’s underlying mechanisms advances and detection technologies improve, its potential for applications in complex wound healing and skin regeneration is expected to become increasingly important.

#### 3.4.5 Skin cosmetics and aging

Lactate’s interaction with mitochondria and ROS is a key mechanism in skin aging and warrants further investigation (150). Lactate has been shown to stimulate both the expression of CD44 and hyaluronan in H8 27 human dermal fibroblasts (150). As a potent moisturizer and a direct inhibitor of melanin synthesis, lactate is increasingly used in the cosmetic industry, particularly for brightening and moisturizing purposes (134, 154). Topical application of lactate has been reported to improve skin smoothness and reduce mild wrinkling caused by photoaging (134). Chemical peeling with lactate is the widely employed treatment for fine lines, photoaging, and pigmentary disorders (155–157). Recently, poly-lactic acids have been clinically utilized to address skin aging, with poly-D,L-lactic acid (PDLLA) shown to stimulate collagen synthesis and even promote angiogenesis in the skin of aged mice models (158, 159). The lactate released by poly-L-lactic acid(PLLA) can be transported into fibroblasts, promoting lactylation at lysine 752 of latent-transforming growth factor beta-binding protein 1(LTBP1) through a lysine acetyltransferase 8 (KAT8)-dependent mechanism (39). This, in turn, increases the protein levels of collagen I and collagen III in fibroblasts (39).

## 4 Conclusions and perspectives

This review began by exploring the fundamental characteristics of lactate synthesis and turnover in the skin, followed by a systematic analysis of its role in regulating the skin barrier and inflammation. We also discussed the potential applications of lactate in the treatment of skin disorders. Lactate, a byproduct of glycolysis, has attracted attention for its dual effects on skin health. On the one hand, it plays a pivotal role in maintaining skin homeostasis by regulating pH levels, enhancing the skin barrier, and suppressing inflammation. These beneficial effects are critical for preserving the skin’s physical and chemical defenses. On the other hand, excessive lactate production can act as a signaling molecule, modulating immune responses and contributing to the pathogenesis of inflammatory skin diseases and tumors. The balance between lactate’s pro-inflammatory and anti-inflammatory effects is complex and context-dependent, warranting further investigation through both basic and clinical research.

Despite significant advances in elucidating lactate’s functions in the skin, several key knowledge gaps remain. Lactate-related receptors, such as GPR81, play crucial roles in inflammation regulation, lipid synthesis, and other physiological and pathological processes. However, the precise roles of lactate-related receptors in regulating skin physiology—such as barrier function and immune responses—and in skin pathology, particularly in inflammatory diseases, are still not fully understood and require further exploration. Similarly, the involvement of lactate in epigenetic modifications, which may influence skin health and disease progression, represents another area in need of deeper investigation. Experimental strategies to address this issue include the utilization of immunoprecipitation mass spectrometry (IP-MS) and 4D label-free technique to analyze lactate-related alterations in chromatin accessibility and histone lactylation patterns, as well as exploring the role of these epigenetic modifications in immune regulation and inflammation.

Recent studies have highlighted a shift towards metabolic reprogramming in the study of inflammatory skin diseases, with metabolic pathway targeting emerging as a promising therapeutic strategy. Emerging evidence suggests that inhibiting key enzymes or transporters involved in lactate production and transport may offer therapeutic benefits. For instance, inhibiting LDH with compounds like oxamate reduces lactate production, and suppresses tumor invasion, and metastasis (160). Similarly, blocking lactate transport using MCT inhibitors, such as syrosingopine, inhibits psoriasis in imiquimod-treated mice models (8). Future research should prioritize the optimization of these strategies, which includes the development of specific inhibitors for LDH and MCT, as well as investigating their potential synergistic effects when combined with existing therapies. Additionally, it is essential to examine the interplay between lactate metabolism and other metabolic pathways, such as oxidative phosphorylation and lipid metabolism, to uncover possible synergistic interactions. In summary, the advancement of targeted therapies that modulate lactate-related receptors or regulate lactate production may offer significant promise in restoring skin health while minimizing adverse effects. Personalized treatment approaches that consider individual metabolic variations could further enhance the efficacy of lactate-based therapies.
